# "All Sorts and Conditions" of Women

**Published:** 1889-02-23

**Authors:** 


### The Doctor's Pulpit.

### "ALL SORTS AND CONDITIONS" OF WOMEN.

### When should Girls Marry?

When should girls marry? If this question were asked of Mr. *Punch*, and if he is still of the mind he was in not very many years ago, he would probably answer, "Never"! Therein we should entirely and absolutely disagree with him. The majority of ladies, at any rate as yet, we feel sure, are on our side. What they may be when the seed of Girton and similar institutions has had time to bring forth fruit, it is impossible to say. A famous ecclesiastic of the Reformation period declared that the "old Adam was too strong for young Melancthon"; and it is more than possible that, Girton notwithstanding, marriage will not lose its popularity for many generations to come. "Marriage," said the Scotch divine, as he was preparing to unite a young couple, "Marriage is a blessing to few, a curse to many, and a trouble to all: do ye venture?" They "ventured" in spite of the portentous warning; and all the facts of the world's history favour the opinion that young couples—and old ones too, for the matter of that—will continue to "venture" to the end of time.

The world and many of its institutions have distinctly improved during the six thousand years of historic time. People do not quite take the same views of many questions that their ancestors took. It would be considered strange, for example, if a man of fashion were to insist upon eating raw meat with his fingers, instead of cooked meat with a knife and fork; or if he were to adorn himself with a wild beast's skin around his loins instead of wearing a properly-cut suit of the tailor's manufacture. Similarly, a lady would be held passing strange if she could write no more elegantly than the ladies of Harold's time, or could spell with no more correctness than those of the period of Queen Elizabeth. Some practical advance, it will be admitted, has been made "all along the line." If we have come to more rational ideas and methods in many of the details of life, it is worth consideration what position we occupy with respect to some of the oldest and most universal institutions of man and of society. Curious people might ask the question—At what age did our ancestors marry, and under what circumstances of haste or of deliberation? There is abundant evidence to show that among some ancient peoples the marriage relation was held to be one of great seriousness, and was not entered upon without the most careful consideration on the part both of the parents and the parties more immediately concerned. Those persons, of either sex, who are ready to marry, at a moment's notice, a man or a woman of whom they know little or nothing, except by appearances and outward circumstances, prove the exceeding inconsequence of their own mind and character. Not only are they intellectually contemptible, but their moral and judicial weight are of no account. Married people soon find out the nature of the step they have taken, whatever inexperienced boys or girls may think about it. There are many, it is to be feared, who are boys and girls in sense even after they have reached so mature an age as thirty or thirty-five. They are prepared to enter upon married life with a heart as light and a mind as careless as if they were no more than seventeen.

The question of the period of marriage will be discussed here mainly from the physiological stand point. Other things being equal, physiology gives its verdict in favour of a fairly early marriage. Probably the best time for the average civilised woman would be any age between twenty-four and thirty-six. It is not said that no woman should marry earlier or later than either of these ages; but youth and health and vigour are ordinarily at their highest perfection between those two periods. Very early marriages are seldom desirable for girls, and that for many reasons. The brain is immature, the reason is feeble, and the character unformed. The considerations which would prompt a girl to marry at seventeen would in many cases have very little weight with her at twenty-four. At seventeen she is a child, at twenty-four a woman. Where a girl has intelligent parents, the seven years between seventeen and twenty-four are the period when both mind and body are most amenable to wise discipline, and best repay the thought and toil devoted to their development. Before seventeen few girls have learnt to understand what life is, what discipline is, what duty is. They cannot value what is best, either in the father's wisdom or in the mother's tenderness. When married at that childish period they are like young recruits taken fresh from the farm and the workshop, and hurried off to a long campaign without any period of preliminary drill and training; or like a schoolboy removed from school to a curacy without being sent to the university or to a theological hall. Who can help grieving over a child-wife, especially if she have children, and a husband who is an inexperienced, and possibly exacting boy-man. The ardour of his love soon cools; the visionary bliss of her poetical imagination vanishes like the summer mist; there is nothing left but disappointment and wonder that what promised to be so beautiful and long a day should have clouded over almost before sunrise.

Girl marriages are markedly unfavourable to after-health. Very decidedly is this the case at highly-civilised periods. Even in unmarried young women appetite is often poor and digestion feeble. All too little blood is made for the full and energetic development of body and brain. Marriage in the very young often makes appetite and digestion poorer still; and new demands upon the blood supply leave little or none for the uncompleted and unestablished organs of action and thought. Such marriages should never be permitted, except in circumstances where the best of food, the purest of air, and the easiest and most prosperous of conditions are assured. Even then, it is better, if it can be brought about, to wait until at least twenty or twenty-one is passed. Among the poor, who live in miserable houses and cannot obtain any but the very poorest food, early marriages are absolutely ruinous. The child-wife becomes mostly a bloodless, healthless, spiritless, and mindless creature, unfit in every particular for the life-struggle which is usually the lot of the labouring class. She is not able either to work or think, and sinks generally from one degree of helplessness to another, until at forty-five or fifty she is an old and imbecile woman. In London and other large towns such women are numbered by tens of thousands. Among this class, at any rate, marriage should be prohibited by law until a suitable age and favourable circumstances justify the experiment..

The children of very young parents inherit the defective constitution of parental immaturity. Of course people who have no brains make very little of a fact of this kind. Their mental wings are not strong enough to carry them on such a flight as is here proposed to them. To ask them to look a few years ahead, and to think of consequences which may then be in evidence, is as purposeless as it would be to invite them to a discussion on political economy, or to a consideration of the Epistle to the Romans. But we do not write for fools, but for those who have both judgment and the sense of duty, and who are minded to walk in the right way when that way is pointed out to them. Heredity is a great fact. As is the seed, so will be the tree and its fruit; and as are the parents, so in most essential particulars must be the children and their life and deeds. If a wife be very young, it is better that her husband be a man of mature years, although, of course, it is not desirable that he be old. It is most interesting and instructive, in the case of large families, to note the different physical and mental peculiarities of the children, and to compare them with the peculiar conditions of health and character which predominated in one or both parents before their birth. One child will be excitable, another stolid; one calculating, another careless; one full of robust health, another drooping; one tender and affectionate, another selfish and cold. These all reflect different parental conditions. How frequently it happens that the eldest child of a family will look fine and promising in babyhood, grow rapidly to a splendid maturity, and then gradually sink and die of some wasting disease. The ultimate cause is probably often to be found in the youth and immaturity of one or both parents. The subject opens out a large field, but our space is exhausted. It is not too much to say that hardly any question in practical physiological science is of more importance, or will better repay wide and thorough consideration, than that of marriage, and the fittest time for it to be entered upon.

(*To be continued.*)

